# Challenges of infant nutrition research: a commentary

**DOI:** 10.1186/s12937-016-0162-0

**Published:** 2016-04-22

**Authors:** Alan S. Ryan, William W. Hay

**Affiliations:** 1 Clinical Research Consulting, 9809 Halston Manor, Boynton Beach, FL 33473 USA; 2 Perinatal Research Center, University of Colorado School of Medicine, Anschutz Medical Campus, Mail Stop F441, 13243 East 23rd Avenue, Aurora, CO 80045 USA

**Keywords:** Breastfeeding, Infant formula, Health benefits, Public health policy

## Abstract

Considerable advances have been made in the field of infant feeding research. The last few decades have witnessed the expansion in the number of studies on the composition and benefits of human milk. The practice of breastfeeding and use of human milk represent today’s reference standards for infant feeding and nutrition. Additional research regarding the benefits of breastfeeding is needed to determine which factors in human milk and in the act of breastfeeding itself, singly or in combination, are most important for producing the beneficial effects on infant growth, body composition, and neurodevelopmental outcome. We examine evidence that breastfeeding confers health benefits and offer suggestions on how best to interpret the data and present it to the public. We also describe some examples of well-designed infant nutrition studies that provide useful and clinically meaningful data regarding infant feeding, growth, and development. Because not all mothers choose to breastfeed or can breastfeed, other appropriate feeding options should be subjected to critical review to help establish how infant formula and bottle feeding can confer benefits similar to those of human milk and the act of breastfeeding. We conclude with the overarching point that the goal of infant feeding research is to promote optimal infant growth and development. Since parents/families may take different paths to feeding their infants, it is fundamental that health professionals understand how best to interpret research studies and their findings to support optimal infant growth and development.

## Introduction

In 2020, the U.S. Dietary Guidelines for Americans will for the first time include recommendations for nutrition of healthy infants and young children, as mandated by the Agricultural Act of 2014 [1]. The review program (B-24) that will eventually lead to the full integration of nutritional needs of infants and children from birth to 24 months of age into future U.S. Dietary Guidelines has been established [2]. This creates an unprecedented opportunity to give parents and family members practical guidelines on how best to meet their children’s nutritional needs during a critical time of growth and development. It also brings to the fore the challenges of developing science-based recommendations for nutrition of infants and young children. As an anthropologist and neonatologist, we provide the following commentary on the unique history and complexities of infant nutrition research. We examine the weight of evidence linking human milk and the act of breastfeeding to specific health benefits, but also describe some of the inherent limitations of infant feeding research involving both human milk and infant formulas. Finally, we describe some examples of recent well-designed infant nutrition studies that provide useful and clinically meaningful data regarding infant feeding, growth, and development.

### Historical developments

Over a century ago, the proper feeding and care of infants led to the development of infant nutrition research, and to pediatrics as a medical specialty [3]. Among the most important advances in infant feeding research was the understanding of the composition of human milk and the benefits that human milk and breastfeeding provide to the growing infant. The practice of breastfeeding and use of human milk remain as the recommended and reference standards for infant feeding and nutrition [4].

In the last 50 years, the number of studies on the composition of human milk has increased, in part because of research focused on making infant formulas more similar to human milk, particularly with respect to composition, nutritional value, tolerance, and performance [3]. Many of the early studies on infant feeding focused on the role of different nutrients and their “metabolic balance” in normal infants, thus providing greater insight into the requirements for optimal growth [5–8]. Now, we know that body composition and neurodevelopmental and physical function are just as important as weight gain alone for determining optimal growth and development.

Today, a variety of infant formulas are available worldwide. Infant formulas are available for preterm infants, full-term infants, and toddlers as well as specialized formulas for those infants and children with selected inborn errors of metabolism. The compositions of these formulas vary greatly depending on the nutritional needs of the targeted infant population. The required essential nutrients included in various infant formulas are provided in the global standards established by the Codex Alimentarius Commission in 1981, and revised over the years [9]. This standard also includes a list of food additives that are allowed to be added. Quality control measures such as labeling, packaging, contaminants and hygiene are also specified. In the United States, standards for infant formula are the responsibility of the U.S. Food and Drug Administration (FDA) [10]. The U.S. Code of Federal Regulations Title 21, Part 106 specifies infant formula quality control procedures and Part 107 lists the nutrient requirements and other rules concerning labeling for infant formulas. Not surprisingly, the quality and safety standards for infant formulas are extremely high, exceeding most requirements for other food products [10]. In the European Union, the legislation on the composition of infant formula and follow-on formulas was adopted in 2006 and at the time of this writing is being revised [11].

### Complexities of infant nutrition research

The development of sophisticated analytical techniques and ability to identify and investigate the effects of certain nutrients at a cellular level led to growth in the number of known compounds in human milk. These components include living cells, hormones, active enzymes, immunoglobulins, and a variety of bioactive compounds and compounds with unique molecular structures [12]. With this knowledge, it became increasingly apparent that concentrations and types of nutrients in infant formulas cannot always match exactly those in human milk.

Studies have shown that some nutrients in human milk, such as fat, protein, and the long-chain polyunsaturated omega-3 fatty acid (LCPUFA), docosahexaenoic acid (DHA), vary from the beginning to the end of each breastfeeding event, between the stages of lactation, and as a result of different maternal diets [13]. The actual contribution of these variations or many of the other milk components to the nutrition provided by human milk, however, has not been completely studied to determine their specific roles in affecting infant nutritional outcomes. In contrast, new ingredients that are added to infant formulas must be shown to be safe and provide important clinical benefits based on extensive and carefully conducted clinical trials. Furthermore, even in clinical practice, comparisons between human milk and infant formula feeding are difficult to determine. Intake volumes and exact composition and thus delivery of nutrients from infant formula feeding can be known quite precisely. In contrast, lack of clinically available milk analyzers to measure even the gross nutrient components in human milk (protein, in particular, but also lipids and carbohydrates) and imprecise determination of human milk feeding volume (weighing the infant before and after each feeding is beyond the capacity of most parents, except for short term and specific indications) limit assessment of what nutrition an infant actually receives from human milk feeding. Thus, comparisons between feeding outcomes of human milk and infant formulas often have lacked a sufficiently common research basis to determine whether infant formulas are meeting the many unique, but incompletely understood compositional benefits of human milk.

Recent research has demonstrated that infant nutrition during critical windows in early development, both pre- and postnatal, has the potential for producing lifelong impacts on health and disease in childhood, adolescence, and adult life [14–16]. The concept that the adequacy or deficiency of a nutrient at a critical period of development could influence or “program” a health or disease outcome has important implications for individuals and public health [16].

There has been an explosion of media attention on infant feeding research. Even though popularization of such studies through social media now is rampant, many of the media reports do not represent easy to understand explanations of results. Not uncommonly, they also frequently lack rational scientific underpinnings (such as real data from randomized, controlled investigations) and fail to clearly show how new research has led to important new approaches to nutrition, some of which are at odds with previous recommendations. Thus, public health recommendations often are inconsistent and inadequately supported by scientific evidence, and in many cases may be misunderstood by new parents. Most importantly, parents often find it difficult to understand how modern infant nutrition research applies to meeting the practical, day-to-day nutritional needs of their growing infant. Similar confusion exists among health professionals [17].

### Problems with definitions of infant feeding and potential self-reporting bias

One of the problems associated with infant feeding studies is that researchers use different definitions of breastfeeding. For example, the “act of breastfeeding” typically refers to the mother’s behavioral interaction with her baby during feeding. Such behavioral interactions include many types of contact (gentle to rough), temperature and heat transfer, enface engagement, verbal communication, and duration of contact, among others, as well as transfer of mother’s microbiota. Behavioral interaction during feeding also can involve other people—father, siblings, other relatives, friends, all of whom can provide different or additional effects on infant development that could influence effects specific to milk or other foods. When considering the actual food ingested by the infant, many infant feeding studies use “breastfeeding” as the primary analytical variable while other studies use “exclusive breastfeeding” [4, 18]. The term “breastfeeding” includes “infants fed human milk or a combination of human milk and formula or cow’s milk”, and perhaps other foods too [19]. “Exclusive breastfeeding”, on the other hand, typically implies the feeding of human milk alone, although some studies also include the feeding of other foods that are not human milk substitutes. Thus, it is difficult to draw conclusions regarding the benefits of “exclusive breastfeeding”, if that is the variable of interest, because the majority of studies do not control for the confounding effects of the additional non-human milk food items included in the infants’ diet. Such limitations in defining degrees of breastfeeding also fail to determine just how much breastfeeding (or human milk feeding if donor milk is used) actually produces defined outcomes.

Dietary surveys of infant feeding practices, especially those conducted orally (e.g., by telephone), are often affected by self-reporting bias, when participants tend to under-report behaviors that are perceived to be inappropriate by researchers and over-report behaviors viewed as appropriate [20]. The act of breastfeeding easily can be placed in the category of a behavior perceived to be “appropriate.” It seems likely that the practice of breastfeeding obtained from a telephone survey may not be accurately reported and as a result may confound the results.

### Infant feeding studies: problems related to study design and weak associations

Infant feeding studies often focus on growth and development, a specific disease, medical condition, or endpoint (e.g., IQ or vision) and can be separated into two different categories: 1) those that show areas of research that are promising and require more resources and time to pursue, but do not provide conclusive evidence, and 2) those that provide more concrete evidence [21]. Confusion arises when the results presented are preliminary or are obtained from a subset of a few studies without a balanced presentation of all the data [21]. The types of studies that are commonly used to evaluate the effects of food and/or food components on infant health and growth and development are described in Table 1. These types of studies also are used for a variety of other purposes, including testing the efficacy and safety of pharmaceutical products.

**Table 1:** Advantages and Limitations of Various Types of Nutrition Studies

Type of Study/Description	Advantages	Limitations
Cell culture – in vitro Food item is placed in cells or other tissues in culture conditions.	Help determine mechanisms of action. Provide clues for further investigation to define mechanisms responsible for how food components interact with host cells.	What occurs in cells may be different from what occurs in human body. Not conclusive.
Animal – in vivo Food item is fed to laboratory animals (e.g. rat, mouse, guinea pig, rabbit). Tests the effects of food on certain diseases, physiological conditions, and behaviors	Can be tightly controlled for testing the metabolism, specificity, and reproducibility of the effect of a certain food component. Tests the toxicity and safety of food components added to the diet.	Humans differ from animals in many aspects of their physiology, such as food digestion, nutrient absorption, genetics, lifestyle, etc. Not conclusive.
Case studies One individual’s experience with food or a food component is documented.	Help determine how certain food/components may affect clinical conditions or disorders in humans. Focus on social, psychological, or medical conditions. Provide clues for further investigation.	Not scientifically rigorous – only one person’s experience. Not conclusive but is more evidence-building.
Epidemiological/observational Groups of subjects, typically living in one geographical area, who have developed a disease or condition are compared with a similar sample of subjects who have not developed the disease.	Address whether a certain food/component could cause a disease but not whether it did cause a disease.	Memory recall is often used to assess how a food/component might have affected an individual – long-term memory recall may not be accurate. Regionally biased – differences observed between regions may be related to different dietary of cultural preferences or genetic differences. Not conclusive but is more evidence-building.
Prospective cohort studies A large group of individuals with a similar background who are healthy when the study begins are followed over time. Diets are assessed at the beginning, during, and end of the study. Eating habits of those who get a disease are compared to those who do not acquire a disease.	Provide clues about the risk/benefits of a given diet, food component or lack of an important vitamin/mineral (e.g., development of iron deficiency and neurodevelopmental impairment) over time.	The controls may not be similar to those who acquire a disease with respect to demographic and health factors. Not conclusive but is more evidence-building.
Randomized, controlled clinical trial (RCT) Evaluates the efficacy of a specific nutritional intervention (e.g., food/component) within a population. Subjects with similar backgrounds are randomly allocated to receive either the test item or placebo.	Represents the “gold standard” for clinical trial methodology. Randomization minimizes allocation bias by balancing both known and unknown social and health factors. Sample sizes are often large to detect subtle differences in treatments, diet or food component when they exist. Provides most conclusive evidence.	Unethical to randomize breastfed infants into an infant formula feeding group.
Meta-analysis Review of the existing scientific literature. Pooled data from several studies are subjected to a statistical meta-analysis.	Meta-analysis of trials provides a more precise estimate of the treatment effect because of the increased sample size and statistical power. Results can be generalized to a larger population. Inconsistency of results across studies can be quantified and analyzed.	The validity of the meta-analysis depends on the quality of the systematic review and studies included in the analysis. If confounding variables are not controlled for in the primary studies, there will be potential bias in the meta-analysis (e.g., differing definitions of breastfeeding).

A problem with all nutrition and infant feeding studies is that the list of possible influential factors is large when each is not studied in isolation using rigorous, randomized experimental study designs, making it difficult to evaluate both short- and long-term effects of any form of infant feeding or nutritional substance used on any one or group of outcomes [17].

The most obvious problem in neonatal nutrition is that while the randomized clinical trial (RCT) is the most scientific and rigorous method to evaluate the efficacy of human milk feeding and the act of breastfeeding on selected outcomes (neurodevelopment, body composition, etc.), it is impossible to conduct an RCT comparing human milk-fed vs. formula-fed infants, because it is unethical to randomly assign infants to a breast-fed or formula-fed treatment group. However, RCTs have been promising in examining the effects of specific ingredients added to infant formula and/or human milk. Often, a different human milk-fed group is used for comparison as a control, although not all studies are careful to select a different comparison group to be as close to the study group as possible in all other factors (e.g., see Ryan et al. [22] for a review of RCTs that considered the nutritional effects of LCPUFAs added to infant formulas and human milk on neurological development). Consequently, such infant feeding research is vulnerable to many potential confounding effects that are left unexamined, such as the different definitions of infant feeding that are used among various infant feeding studies or that different groups are unique in other aspects that are not controlled. It should be kept in mind, however, that the cost of RCTs is typically very expensive. Sometimes, smaller, proof-of-concept studies can initially address some fundamental questions and provide evidence needed to support additional RCTs.

Epidemiological/observational (E/O) studies seem to draw most of the attention from both health professionals and the media. A number of nutritional claims with “strong support” from E/O studies have in fact shown conflicting results when the total body of evidence is considered [17, 23, 24]. As examples, some observational follow-up studies indicated improved cognitive and developmental outcomes in infants whose mothers’ diets were supplemented with omega-3 LCPUFAs, particularly DHA, as the fetal brain accumulates these LCPUFAs rapidly in the second half of pregnancy [25–27], while other follow-up studies did not report improved outcomes [28]. Even a systematic review and meta-analysis of maternal LCPUFA supplementation could not conclusively refute or support supplementation during pregnancy for improving cognitive and visual outcomes of offspring [29]. However, a recent consideration of childhood allergies indicated some potential long-term benefits of LCPUFA supplementation [30, 31].

### Examples of promising infant feeding studies

There are many documented benefits to human milk feeding and the act of breastfeeding (although the research base varies widely in the strengths and weaknesses in study designs, controls, and findings), which can include reduced rates of necrotizing enterocolitis (NEC), respiratory infections and otitis media, sudden infant death syndrome, gastrointestinal infections, atopic diseases, celiac disease, inflammatory bowel disease, diabetes mellitus, leukemia, obesity, and improved neurodevelopmental outcomes, particularly in relation to the duration of breastfeeding [4]. In some cases, it is reasonably clear that such benefits are specific to the milk itself, for example, with reduced rates of NEC, since preterm infants who develop NEC are fed by gavage and not from the breast.

Optimal nutritional management of NEC is of great interest because NEC is the most common and dangerous gastrointestinal medical emergency in premature infants [32]. The type of infant feeding and its relationship to the development of NEC has been the subject of several clinical trials [33–36]. In a large prospective trial (an RCT cannot not be performed in this sensitive population as discussed above), human milk-fed infants developed NEC 6 to 10 times less often than those fed infant formula exclusively and 3 times less often if they were fed a mixture of human milk and infant formula [36].

Systematic reviews of RCTs have indicated that probiotics added to infant formula and/or human milk may reduce the incidence of mortality from NEC and the development of late onset sepsis [37, 38], as well as reducing the time to full enteral feedings that resulted in better weight gain and growth [39]. Probiotics also have been suggested to improve the quality of intestinal mucus, increase gut motility, and limit the production of inflammatory cytokines [33, 39]. Most of the RCTs used combinations of different strains of Bifidobacterium added to infant formula or human milk, making it difficult to determine which of the many different probiotic organisms is most beneficial. Clinical trials are ongoing to determine the most effective preparation of probiotics [40]. The most recent trial in the UK (PiPS Study, or Probiotics in Preterm Infants Study) did not show a benefit to reducing NEC, although it used only one probiotic organism (*Bifidobacterium breve*) [41]. Whether probiotics actually do reduce the rates of NEC and/or late on sepsis remains highly controversial [42], with clinical practice widely variable, even for the use of some of the probiotic organisms that have shown promise [43].

Additional research is needed to identify those attributes of human milk and those related to the act of breastfeeding that underlie improved outcomes. It remains unclear, for example, what it is in human milk that reduces the risk of NEC. It might be nutrient components of milk (DHA, for example, or immunoglobulins, lactoferrin, lysozyme, and various immunonutrients) [44], or it might be the microbiome that comes from the milk or independently from the mother and not the human milk itself [45, 46].

Some human milk oligosaccharides (HMOs) also might have a protective role in preterm infants by decreasing pathogens associated with sepsis and NEC, but other HMOs can increase the composition of abnormal and potentially pathogenic organisms among the intestinal microbiota [47]. There are considerable differences among the HMOs in the milk of mothers of just born preterm infants than later after birth or from term infants or from mature donor milk [48, 49]. HMOs appear to have at least two positive functions: they produce prebiotic activity by stimulating the growth of commensal bacteria in the gut and they provide protection against pathogens [48, 50]. Oligosaccharides are also associated with infant growth and body composition [51]. Studies have suggested that certain growth factors (e.g., epidermal growth factor, a compound found in human milk and not in infant formula) may reduce the risk of NEC by limiting the damage caused by bile acids [52].

Determining such factors is essential to help modify infant formulas to provide the same protective benefits as human milk, since many preterm infants are fed infant formulas, and also because what initially might appear beneficial, on more detailed analysis, could include some adverse components.

Equally compelling would be the consideration of the specific role of the microbiome that is produced from human milk (preterm, term, or donor human milk), infant formulas, and the act of breastfeeding, since interaction of microbes and the human host mucosal immune system likely play major roles in diseases seen in the neonatal intensive care unit such as NEC and late onset sepsis. This interaction also likely relates to subsequent health in terms of susceptibility to allergic and autoimmune diseases and the metabolic syndrome. Another area of research for human milk, infant formulas, and the act of breastfeeding is to determine what constitutes a “normal” intestinal microbiome in the infant, regardless of gestational age at birth or postnatal age. The influence of behavioral factors that affect the microbiome and its functions other than human milk or infant formula and their components may be just as important, particularly for those infants exposed to antibiotics, either ante-or postpartum [53].

It also is fundamental to identify those attributes of the act of breastfeeding that could be important for optimal nutrition of infants and for reducing adverse outcomes as well as improving neurodevelopment. Could skin-to-skin contact even without breastfeeding or human milk feeding be helpful in establishing a healthy microbiome in the infant based on the mother’s microbiome? How might the way the breast-fed infant is held, how often, and for how long at each holding provide important clues for how a mother might adapt her bottle-feeding techniques to more closely mimic what might be beneficial in the breast-fed infant? Could holding and feeding by the father or siblings or other caretakers provide the same benefits for the bottle-fed infant that might be important for outcomes noted among breast-fed infants? Such questions and others should be addressed, not just to help parents improve feeding skills, but also to determine what is beneficial to breastfeeding specifically, since many human milk fed infants are fed expressed milk by bottle. Such outcomes also might benefit those infants fed infant formulas by bottle.

New infant formulas continue to be tested in an attempt to achieve similar benefits provided by human milk. For example, to prevent and treat atopic diseases and allergies during infancy, partially hydrolyzed infant formulas are becoming increasingly used worldwide [54]. A review of the literature to determine whether these infant formulas may be recommended for feeding all infants, if breastfeeding is not possible, revealed that adverse effects on health were not apparent, but data about potential benefit or risk of specific long-term outcomes, particularly those referring to immune, metabolic and hormonal effects, were nonexistant [54].

## Recommendations and conclusions

When public health recommendations regarding infant feeding (both breastfeeding and infant formula feeding) are disseminated, scientists and public health officials must put the strengths and limitations of the existing research into proper perspective. There needs to be a commitment to faithful reporting, a balance of data presentation, and a description of evidence in non-misleading ways [55]. New study design techniques, such as sibling-pair comparisons in which one sibling is breast-fed while another is not, are being used that may help account for the selection bias that typically hinders efforts to measure more precisely the effects of any one or mixed type of infant feeding [56, 57]. Even this sort of study design, however, does not control for why a mother would chose to feed two infants differently. To help distinguish the act of feeding from its use to provide food, other studies should investigate the effects of administering human milk vs. infant formulas through gavage tubes or via bottles on infant development [14], particularly in preterm infants in the neonatal intensive care unit, where the qualities of what is fed through gavage tubes or by bottles are separated from those of the mother providing human milk by breastfeeding (including specifically the use of donor milk, which is pooled from many women of different backgrounds and conditions and environments). Specific ingredients also have been added to infant formulas and human milk to measure their effects on a variety of outcomes [58]. The impact of variation in human milk composition also needs to be further explored. Unfortunately in the U.S., the FDA has not yet licensed milk analyzers for clinical use, making it difficult to know what an infant who is fed milk, of any kind, actually receives in terms of nutrient components and thus how to optimize nutrition of an infant who might need more or less of any one component of milk.

Providing human milk (as a food) cannot be easily distinguished from the act of breastfeeding any more than infant formula and table food feeding can be considered separately from the behavior of those doing the feeding and the many environmental conditions that are involved in feeding infants. The act of breastfeeding may represent an orientation toward a parenting style that in itself has some positive intrinsic value relative to health [17]. Conscientious feeding of infant formulas and table foods also can have specific benefits.

The goal of infant feeding research, above all, is to promote optimal infant growth, body composition, and development. In evaluating the scientific evidence base for developing infant and young child nutrition recommendations, it is important to consider the many inherent limitations of various methodologies and study designs. Feeding recommendations need to be informative, yet practical, and balanced to help address the real world decisions today’s diverse families and caregivers must make when it comes to feeding infants and young children.

## Abbreviations

DHA: docosahexaenoic acid
FDA: U.S. Food and Drug Administration
HMO: human milk oligosaccharides
LCPUFA: long-chain polyunsaturated fatty acids
NEC: necrotizing enterocolitis
RCT: randomized, controlled trial
